# Pan-cancer analysis of disulfidptosis with potential implications in prognosis, immune microenvironment, and drug resistance in human cancer

**DOI:** 10.18632/aging.205993

**Published:** 2024-07-03

**Authors:** Fobao Lai, Wanrong Zheng, Chengqian Zhong, Zhiyong Chen

**Affiliations:** 1 Department of Oncology, Longyan First Affiliated Hospital of Fujian Medical University, Longyan 364000, Fujian, China; 2 College of Medical Nursing, Minxi Vocational and Technical College, Longyan 364000, Fujian, China; 3 Department of Digestive Endoscopy Center, Longyan First Affiliated Hospital of Fujian Medical University, Longyan 364000, Fujian, China

**Keywords:** disulfidptosis, tumor immunity, tumor microenvironment, drug resistance

## Abstract

To get a systematic assessment of disulfidptosis-related genes across human cancers and explore the predictive role of disulfidptosis in cancer drug sensitivity. We developed a score-level model to quantify the level of disulfidptosis in 33 human cancers using TCGA data. The mRNA expression and protein levels of disulfidptosis-related genes in human cancer cells and tissues were detected and retrieved from the Human Protein Atlas. Multiomics bioinformatic analyses were performed to evaluate disulfidptosis-related gene characteristics as well as the effect of disulfidptosis on the cancer immune microenvironment and drug resistance. Thirty cancers showed significantly different expression levels of disulfidptosis-related genes between normal and tumor samples. The mRNA expression and protein level of disulfidptosis-related genes were consistent with TCGA databases in lung cancer and hepatocellular carcinoma. We also found that altered levels of the disulfidptosis score expression were usually related to patient prognosis, and high expression of disulfidptosis-related genes was associated with drug resistance in different cancer types. Our study illustrates the characterization of disulfidptosis in multiple cancer types and highlights its potential value as a predictive biomarker of drug response, which can pave the way for further investigation of the prognostic and therapeutic potential of disulfidptosis.

## INTRODUCTION

Disulfideptosis is a novel form of controlled cell death that has recently been described [1]. During disulfideptosis, intracellular disulfide molecules accumulate abnormally, and actin cytoskeleton proteins fail to form disulfide bonds, resulting in cell death. Additionally, this study showed that GLUT inhibition-induced disulfidptosis may be a useful therapeutic approach for treating SLC7A11^high^ tumors, which are frequently observed in human malignancies [1]. However, the expression pattern and prognostic significance of disulfidptosis, particularly the relationship between immune infiltration in pan-cancer and the expression levels of genes related to disulfidptosis, remain largely unknown.

Solute carrier family 7 member 11 (SLC7A11, also commonly known as xCT) and solute carrier family 3 member 2 (SLC3A2; also known as CD98 or 4F2hc) are catalytic components of system Xc, which transport extracellular cystine into cells for glutathione production [2] and have been identified as one of the major controllers of disulfidptosis. The cystine transporter system, xc-, is mostly responsible for bringing in cystine, which is then transformed into cysteine in the cytosol through a reduction reaction that consumes NADPH and is used to produce glutathione (along with other biomolecules) in the majority of cancer cells [3].

Unrelated to ATP depletion or the generation of cystine crystals, disulfidptosis is a new type of cell death that has only recently been identified when high SLC7A11 expression and glucose starvation are combined. Notably, evidence suggests that SLC7A11 is a key oncogenic protein that influences malignant cancer behavior, tumor microenvironment, immune system, cancer-associated syndromes, and therapeutic susceptibility [4–6]. Additionally, SLC7A11 is overexpressed in many different cancer types and is associated with a poor prognosis for patients [4, 7, 8].

SLC3A2, a single transmembrane protein and a key regulator of disulfidptosis, functions as a chaperone to preserve the stability and proper membrane localization of SLC7A11 [9]. Research showed that SLC3A2 promotes cancer cell proliferation and confers a poor prognosis for various cancer types [10, 11]. SLC3A2 can also act as a metabolic switch in lung adenocarcinoma cells to induce macrophage phenotypic reprogramming through arachidonic acid [12]. What’s more, SLC3A2 was involved in the tamoxifen resistance in ER+ breast cancer and the chemoresistance to cisplatin in ovarian cancer cells [13, 14].

The Rac-WRC pathway-generated branched actin network in lamellipodia serves as a primary target for disulfide bonding among actin cytoskeleton proteins, and Rac-WRC-mediated lamellipodia generation promotes disulfidptosis. Nck-associated protein 1 (NCKAP1) is a component of the WAVE regulatory complex (WRC). WRC promotes actin polymerization and lamellipodia development by activating the seven-subunit actin-related protein 2 and 3 (Arp2/3) complex, resulting in the establishment of a branching cortical actin network beneath the plasma membrane. In UMRC6 cells, NCKAP1 deletion reduced glucose-induced disulfide bond formation, as well as F-actin contraction and separation from the plasma membrane. NCKAP1 overexpression promotes disulfidptosis in UMRC6 cells [1]. The WRC catalytic component WAVE-2 stimulates the production of lamellipodia and actin polymerization, both of which are mediated by Arp2/3. Restoration of WT WAVE-2 in WAVE-2-KO UMRC6 cells induced disulfidptosis, but not its VCA mutant, which is unable to connect with Arp2/3 and regulate actin polymerization. It is well known that Rac activates WRC to promote the production of lamellipodia [15, 16], and that disulfidptosis was also induced by overexpressing Rac1-Q61L, a constitutively active mutant version of Rac1 [1]. In addition, UMRC6 cells were more resistant to disulfidptosis when ribophorin I (RPN1), which encodes an N-oligosaccharyl transferase located in the endoplasmic reticulum, was deleted [1].

In the current study, we thoroughly examined the mRNA levels of NCKAP1, SLC7A11, WASF2, RAC1, SLC3A2, and RPN1 as well as the relationships between the mRNA levels of these six genes in pan-cancer. We comprehensively analyzed the association between disulfidptosis and pan-cancer prognosis and investigated its correlation with the immune microenvironment and drug sensitivity. Our research aims to provide more information to better understand the significance of disulfidptosis in various cancers.

## RESULTS

### Disulfidptosis genes differentially expressed between normal and tumor samples in pan-cancer

**Figure 1:**
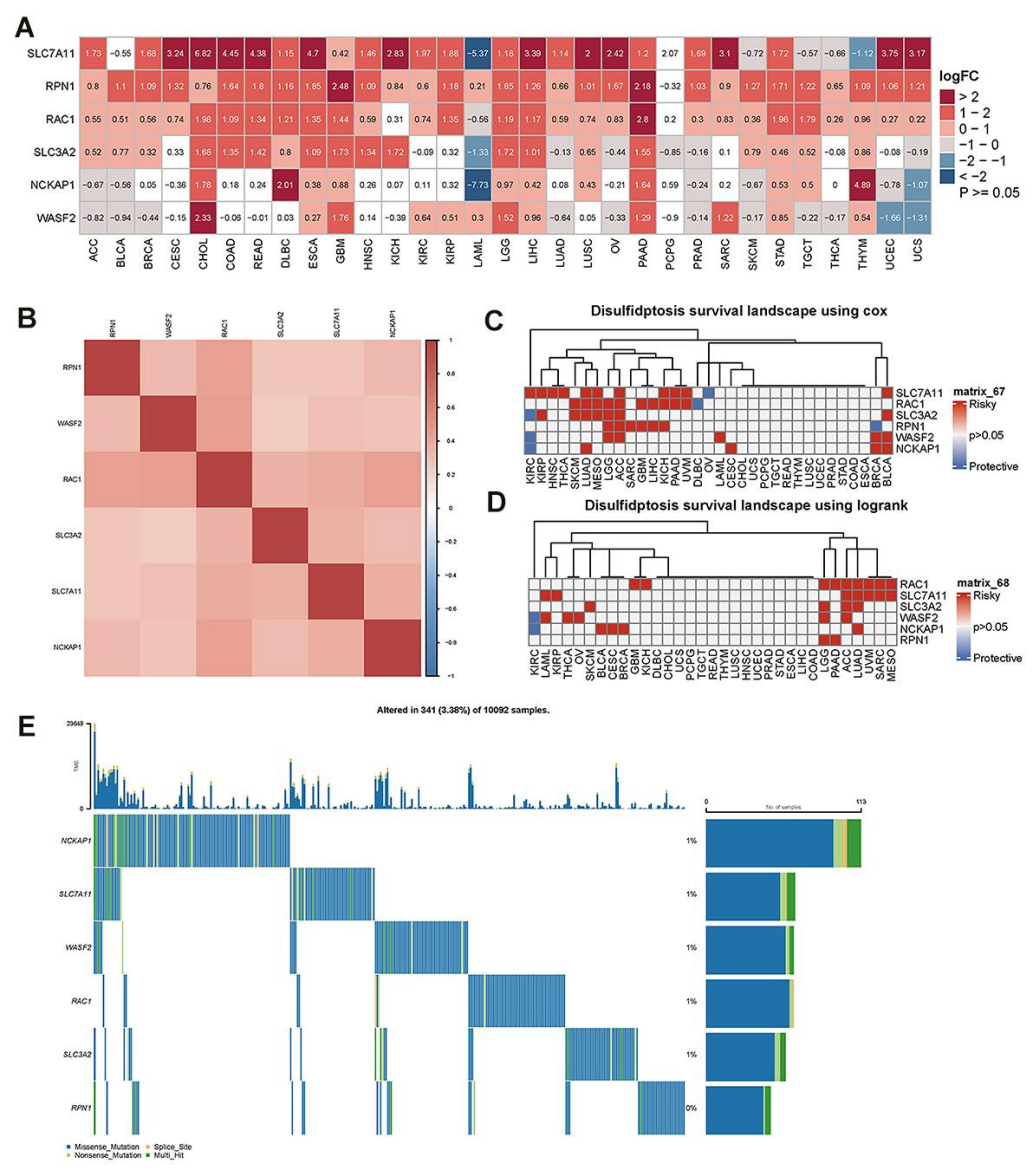
**The difference in disulfidptosis genes expression in pan-cancer and survival analyses.** (**A**) Heatmap showing the difference in disulfidptosis gene expression in 31 cancer types from the TCGA database compared with normal paracancerous tissues from the TCGA and GTEx databases. The red and blue indicate high or low expression (p<0.05), respectively. The white indicates no difference in expression (p≥0.05). (**B**) Heatmap of the correlation between disulfidptosis genes across cancers. Darker colors represent stronger correlations. (**C**) Univariate Cox regression analysis was performed on the disulfidptosis gene in pan-cancer, and the p-value and HR value were extracted and displayed using a heat map (R pheatmap package). The gray indicates p>0.05; for p<0.05, the red indicates HR>1, and the blue indicates HR<1 (R survival and survival packages). (**D**) Kaplan-Meier survival analysis was performed for the disulfidptosis genes in pan-cancer, and a heatmap was performed for display (pheatmap package). p> 0.05 is gray; for p<0.05, the red represents poor prognosis with high expression, and blue represents good prognosis with high expression (R survival and survival packages). (**E**) Genetic alterations of the disulfidptosis gene in pan-cancer.

Using RNA sequencing data from the TCGA and GTEx databases, we evaluated the differential expression of disulfidptosis genes in cancer and paracancerous tissues. Our findings showed that SLC7A11 was more prevalent in 24 different cancer types, particularly CESC, CHOL, COAD, READ, ESCA, KICH, LIHC, LUSC, OV, SARC, UCEC, and UCS. In contrast, LAML, SKCM, TGCT, THCA, and THYM showed reduced expression levels. RPN1 expression has been linked to elevated expression in 30 cancer types, particularly GBM and PAAD. RAC1 was more highly expressed in 28 different types of cancer, especially PAAD. However, lower levels were observed in LAML patients. Additionally, SLC3A2 was more highly expressed in 18 different types of cancers. Lower expression levels were observed in LAML, LUAD, OV, PCPG, PRAD, and UCS. We also investigated the greater levels of NCKAP1 expression in 11 different cancer types, particularly DLBC and THYM. However, NCKAP1 was expressed at lower levels in ACC, BLCA, LAML, PRAD, SKCM, UCEC, and UCS. Additionally, higher WASF2 expression was observed in 12 different cancer types, particularly CHOL. Lower WASF2 expression was observed in 11 different types of cancer, including ACC, BLCA, BRCA, LUAD, OV, PRAD, SKCM, THCA, TGCT, UCS, and UCEC (Figure 1A). The correlation analysis performed by the R-package “corrplot” revealed that the expression levels of the disulfidptosis genes were positively connected with each other, indicating that they may share certain roles or activities (Figure 1B).

To further explore the association of disulfidptosis-related genes with survival, survival analysis of disulfidptosis-related genes was performed using pan-cancer analysis. Univariate Cox regression analyses identified disulfidptosis-related genes as risk factors (HR > 1) in numerous cancers; for example, SLC7A11 was a risk factor for KIRC, KIRP, HNSC, THCA, LUAD, MESO, ACC, KICA, PAAD, UVM, and BLCA (Figure 1C). The prognostic significance of the disulfidptosis-related genes was estimated using Kaplan–Meier survival analysis and compared using log-rank tests. Figure 1D shows that high expression of disulphidptosis-related genes is associated with poor survival in many types of cancers. As a result of this analysis, high expression of RAC1 was associated with the largest number of cancer types, nine of which had poor survival. Taken together, these results demonstrate that disulfideptosis-related genes predict patient survival in certain cancer contexts.

Using the cBioPortal database, we further examined the variation in frequency and forms of disulfideptosis-related genes in 10092 cancer patients (Figure 1E). According to these findings, the rates of gene alterations for NCKAP1, SLC7A11, WASF2, RAC1, and SLC3A2 were approximately 1%. RPN1, however, showed the lowest fluctuation rate, which was close to 0%. In addition, we discovered that the genomic alterations included splice sites, multi-hits, nonsense mutations, and missense mutations. At low mutation frequencies, disulfide ptosis genes are predicted to be mutated in a variety of cancer types.

### The disulfidptosis score was measured using single sample gene set enrichment analysis (ssGSEA) in pan-cancer

**Figure 2:**
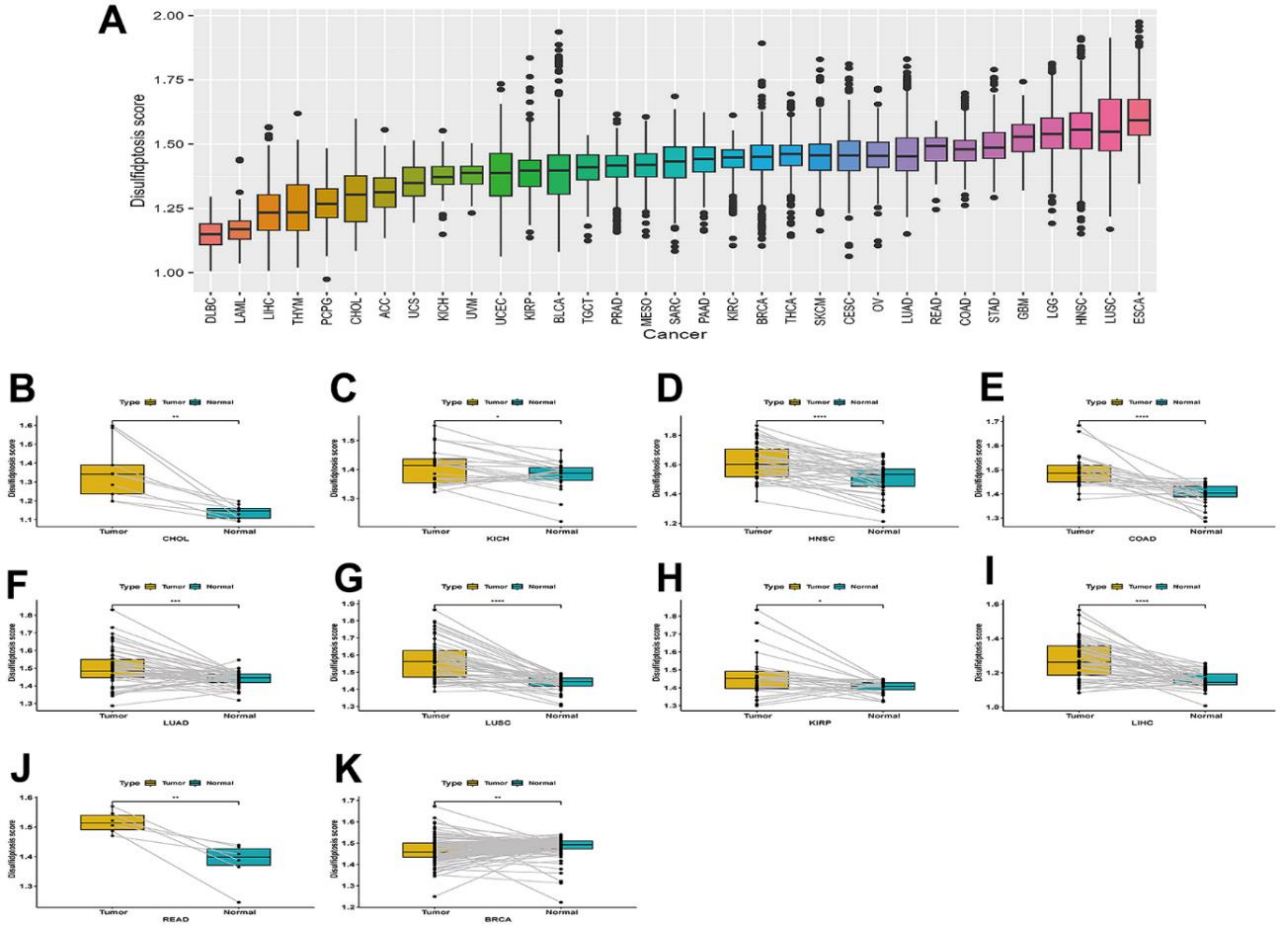
**Boxplot of the disulfidptosis score in 33 tumor types and the difference in the disulfidptosis score of paired cancer and adjacent tissues in different tumors.** (**A**) Boxplot of the disulfidptosis score in 33 tumor types. (**B**–**K**) Disulfidptosis scores varied between paired cancers and adjacent tissues in various cancers. CHOL (**B**), KICH (**C**), HNSC (**D**), COAD (**E**), LUAD (**F**), LUSC (**G**), KIRP (**H**), LIHC (**I**), READ (**J**), and BRCA (**K**).

The disulfidptosis score of TCGA pan-cancer was calculated using the single sample GSEA (ssGSEA) method, and was carried out using the GSVA package in R. Each tumor sample had a disulfidptosis score. A boxplot of the disulfidptosis score in the 33 tumor types is displayed using R ggpot2. The results showed that the disulfidptosis score was lowest in DLBC and highest in ESCA (Figure 2A).

### The difference in the disulfidptosis score of paired cancer and adjacent tissues in various tumors

Disulfidptosis scores were higher in cancer tissues than in normal adjacent tissues in CHOL, KICH, HNSC, COAD, LUAD, LUSC, KIRP, LIHC, and READ Figure 2B–2J. However, the disulfidptosis score was lower in cancer tissues compared to normal-adjacent tissues in BRCA (Figure 2K).

### The mRNA expression and protein level of disulfidptosis-related genes were consistent with the TCGA and GTEx databases

**Figure 3:**
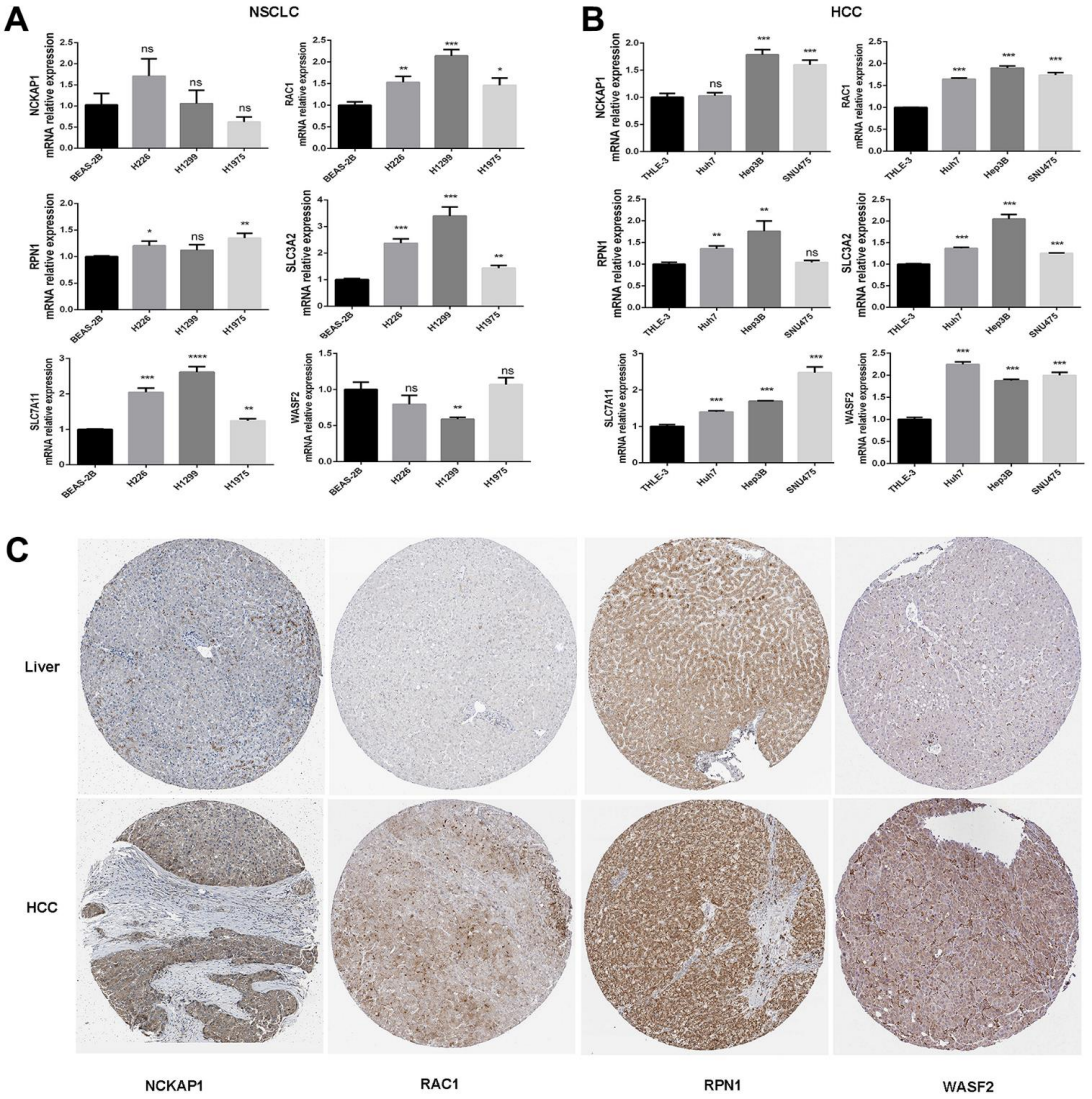
**The mRNA expression and protein levels of disulfidptosis genes were detected.** (**A**) The mRNA expression of human normal lung epithelial cells (BEAS-2B), and lung squamous cell carcinoma cell line (H226), and lung adenocarcinoma cell lines (H1299 and H1975) was detected by RT-PCR. (**B**) The mRNA expression of normal liver epithelial cells (THLE-3) and hepatocellular carcinoma cells (Huh7, Hep3B, and SNU475). (**C**) Comparison of NCKAP1, RAC1, RPN1, and WASF2 immunohistochemistry images in normal and tumor tissues. NCKAP1, RAC1, RPN1, and WASF2 protein expression were significantly higher in LIHC.

In human normal lung epithelial cells and lung cancer cells, the mRNA expression of the disulfidptosis-related genes was evaluated and adjusted to actin. According to the findings, lung squamous cell carcinoma and lung adenocarcinoma cells have higher levels of RAC1, RPN1, and SLC7A11 mRNA than normal lung epithelial cells (Figure 3A). This is in line with the TCGA and GTEx databases. Disulfidptosis genes’ mRNA expression was much higher in hepatocellular carcinoma cells than in normal liver epithelial cells, which was also supported by the TCGA and GTEx databases (Figure 3B). Based on Western-blotting results, disulfidptosis-related gene expression was much higher in hepatocellular carcinoma cells than in normal liver epithelial cells (Supplementary Figure 1).

We then analyzed the IHC results provided by the HPA database; the results of the analysis of the data were consistent with the TCGA and GTEx databases. Normal liver tissue had negative or medium NCKAP1, RAC1, RPN1, and WASF2 IHC staining, while tumor tissues had medium or strong staining (Figure 3C). More patient information about IHC can be seen in Supplementary Figure 2.

### The association between the disulfidptosis score and clinical stages, as well as the prognosis of patients with various tumors

Differences in the disulidptosis scores at different stages of different tumors are shown in Supplementary Figure 3. Examination of tumor stage relevance revealed that the disulfidptosis score significantly increased in tumors at an advanced stage, such that a higher disulfidptosis score was found in stage IV in KICH, LUAD, and STAD.

**Figure 4:**
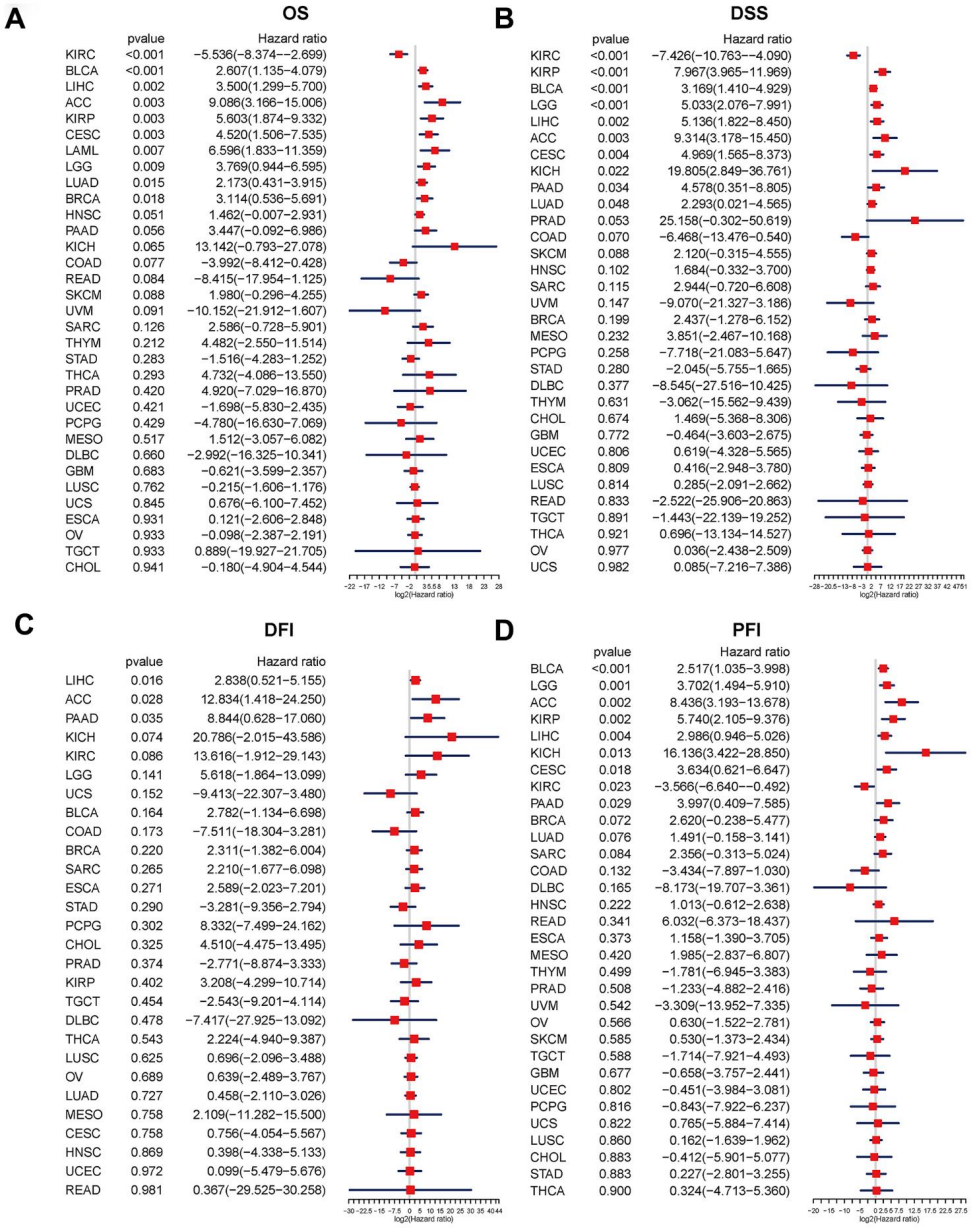
**Relationship between the disulfidptosis score and pan-cancer overall survival (OS), disease-specific survival (DSS), disease-free interval (DFI), and progression-free interval (PFI).** Univariate Cox proportional hazard regression analysis between the disulfidptosis score and pan-cancer OS (**A**), DSS (**B**), DFI (**C**), and PFI (**D**). The abscissa represents the log2 (HR) value. A high-risk factor for cancer with a poor prognosis is one with a hazard ratio > 1. A hazard ratio of <1 is considered to be low-risk for cancer in comparison.

We used the R-language survival package for the Cox proportional hazard regression model analysis and forest mapping, and we claimed that P < 0.05 was significantly related. The disulfidptosis score was linked to a higher advantage of OS and DSS in KIRC, but indicated a poor prognosis in OS for BLCA, LIHC, ACC, KIRP, CESC, LAML, LGG, LUAD, and BRCA, and also predicted a poor prognosis in DSS for KIRP, BLCA, LGG, LIHC, ACC, CESC, KICH, PAAD, and LUAD (Figure 4A, 4B). For LIHC, ACC, and PAAD, the disulfidptosis score indicated a poor prognosis for DFI (Figure 4C). The disulfidptosis score indicated that KIRC would have a favorable prognosis in PFI, but that BLCA, LGG, ACC, KIRP, LIHC, KICH, CESC, and PADD would have a poor prognosis (Figure 4D).

**Figure 5:**
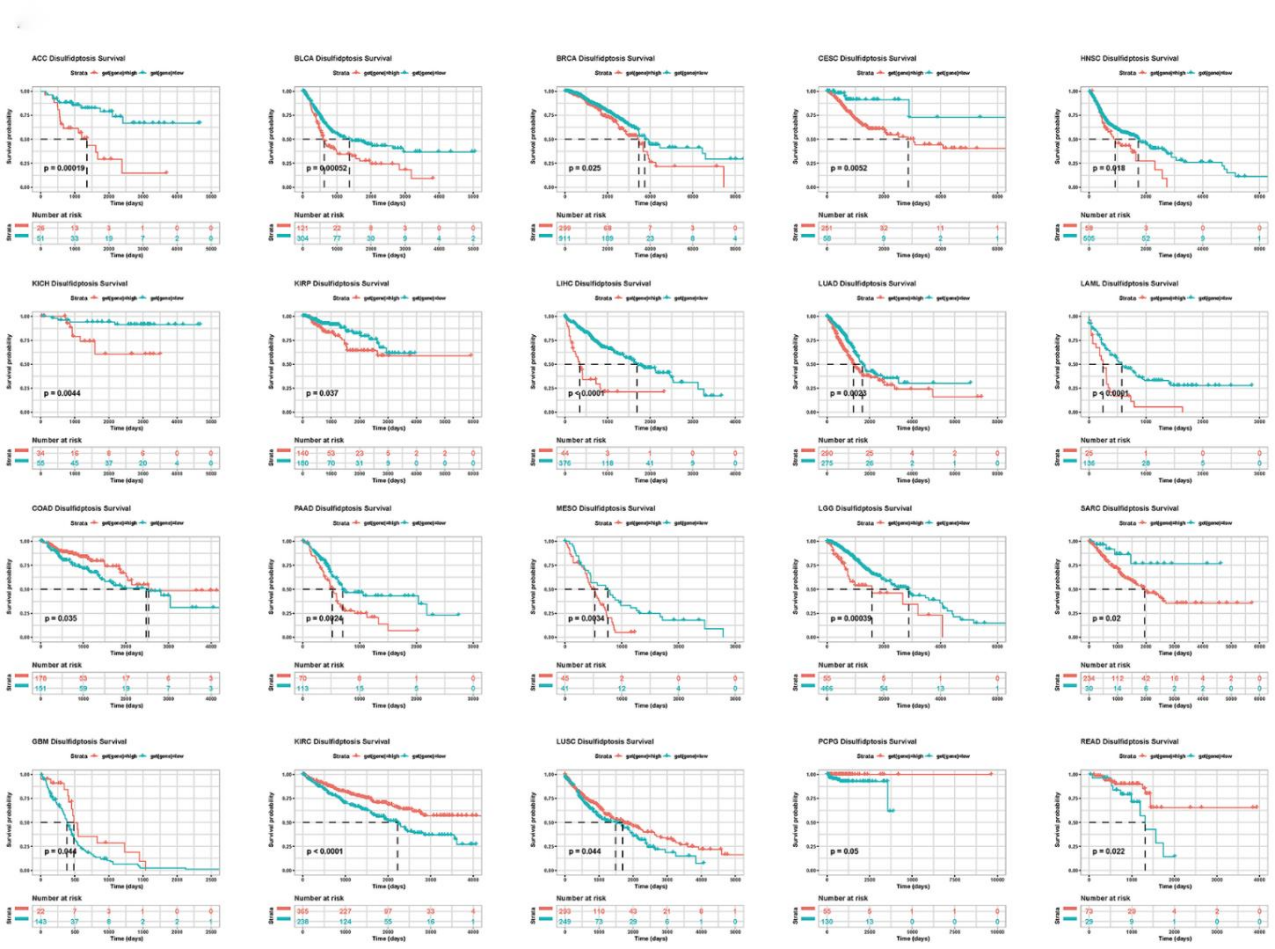
Kaplan-Meier plots of the disulfidptosis score and overall survival.

In addition, we conducted a Kaplan-Meier analysis of the disulfidptosis score in 33 TCGA cancers. Patient survival times and survival statuses were obtained from the UCSC Xena website. We discovered that changes in the disulfidptosis score expression were frequently associated with patient outcomes. In ACC, BLCA, BRCA, CESC, HNSC, KICH, KIRP, LIHC, LUAD, LAML, PAAD, MESO, LGG, and SARC, a high disulfidptosis score was significantly associated with a poor prognosis. A low disulfidptosis score, on the other hand, was associated with a worse prognosis for COAD, GBM, KIRC, LUSC, PCPG, and READ (Figure 5).

### The relationship between disulfidptosis and various pathway scores identified via ssGSEA in 33 cancer types

**Figure 6:**
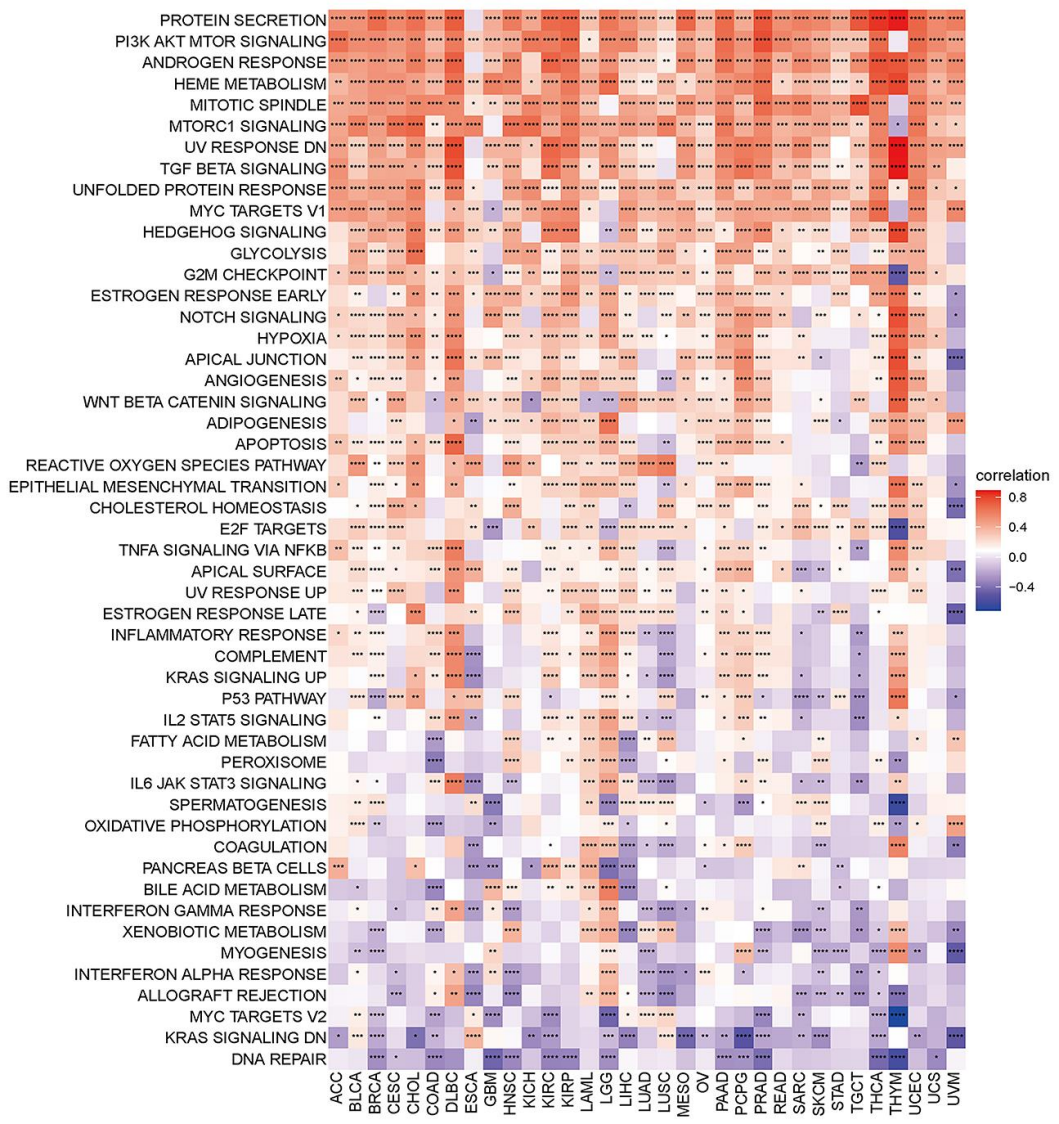
**Heatmap of the correlation between the disulfidptosis score and different pathway scores calculated by ssGSEA in 33 cancer types.** Red represents positive correlation, blue represents negative correlation, and the darker the color, the stronger the correlation. *p< 0.05, ** p< 0.01, and *** p< 0.001, ****p< 0.001.

Gene set variant analysis (GSVA) was performed on all TCGA samples using the HALLMARK pathway database from the molecular signatures database (MSigDB). The R program “corrplot” was used to examine the link between disulfidptosis and various pathway scores in distinct cancer types. This demonstrated that disulfidptosis is significantly associated with immune-related pathways, such as TGF beta signaling, NOTCH signaling, angiogenesis, and WNT beta catenin signaling (Figure 6).

### Relationship between disulfidptosis score and tumor microenvironment in pan-cancer

The above results indicated that the disulfidptosis score was associated with immune-related pathways. Consequently, we investigated the association between the disulfidptosis score and the immune response in a variety of malignancies.

**Figure 7:**
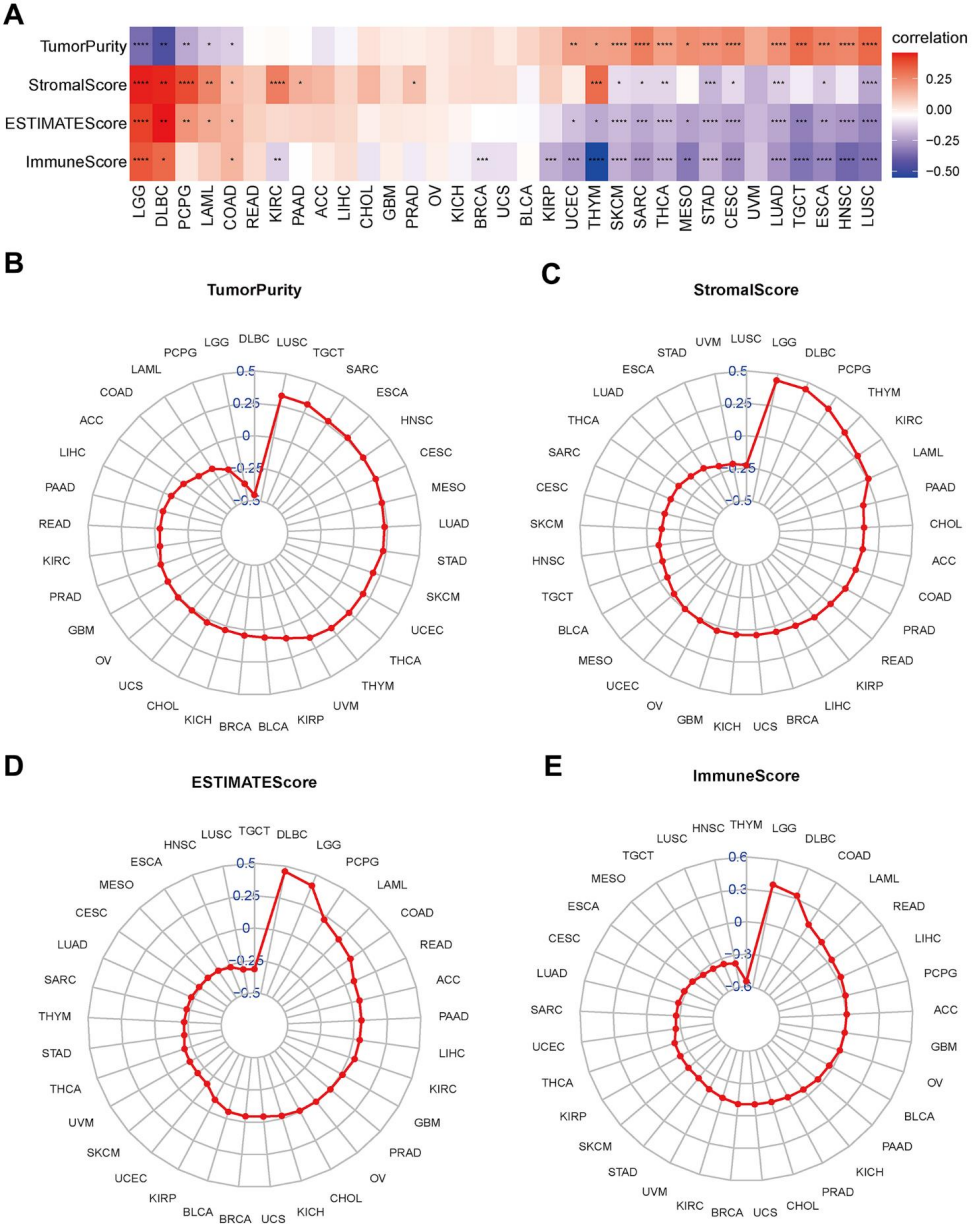
**Association between disulfidptosis score and tumor microenvironment factors in pan-cancer.** (**A**) Heatmap of the correlation between disulfidptosis score and pan-cancer stromal score, immune score, estimate score and tumor purity. Red represents positive correlation, blue represents negative correlation, and the darker the color, the stronger the correlation. *p< 0.05, ** p< 0.01, and *** p< 0.001, ****p< 0.001. (**B**–**E**) Tumor purity (**B**), stromal score (**C**), estimate score (**D**), and immune score (**E**) are illustrated.

According to the tumor microenvironment-related score results, the disulfidptosis score was significantly adversely associated with stromal score, immune, and estimation scores and favorably related to tumor purity (Figure 7). We discovered a clear negative association between immune score and the majority of malignancies. As previously stated, the immune score represents the proportion of infiltrating immune cells in tumor tissues. Subsequently, the relationship between the disulfidptosis score and the level of immune cell infiltration was examined.

**Figure 8:**
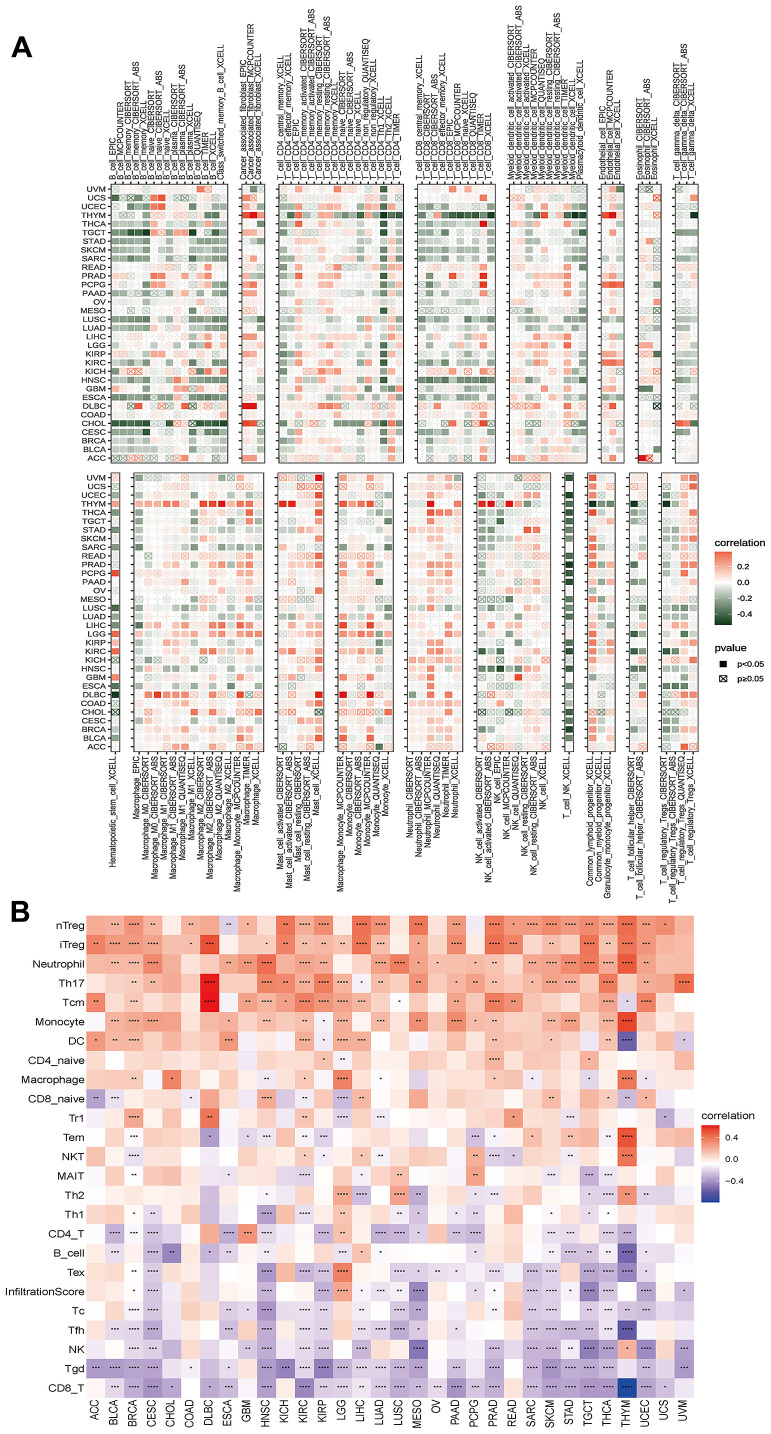
**Analyses of the associations between immune cell infiltration and the disulfidptosis score were performed on a variety of tumor types.** (**A**) The correlation between disulfidptosis score and different immune cell infiltrations using the TIMER2 database in pan-cancer. (**B**) The correlation between disulfidptosis score and different immune cell infiltrations using the ImmuCellAI database in pan-cancer. The red label indicated a positive correlation with the disulfidptosis score, and the dark green label indicated a negative correlation with the disulfidptosis score. *P<0.05, **P<0.01, ***P<0.001, ****P<0.0001.

We found a positive correlation between the disulfidptosis score and various pro-tumoral immune cells, such as Treg cells and tumor-associated neutrophils, and a negative correlation with anti-tumor immune cells, including CD8+ T cells and NK cells. This implies that individuals with high disulfidptosis scores were immunosuppressed (Figure 8).

### Disulfidptosis is linked to the response to anti-cancer drugs or small compounds

**Figure 9:**
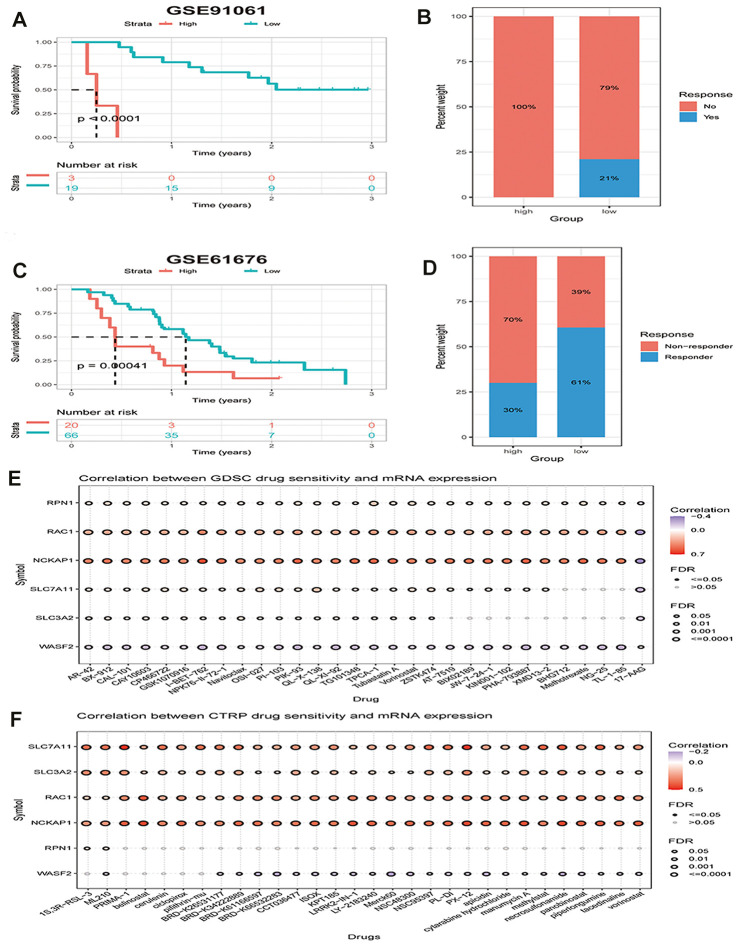
**The disulfidptosis score is associated with response to therapy in multiple cancer types.** (**A**) Kaplan–Meier curves of OS between low and high groups stratified by the disulfidptosis score in the melanoma immunotherapy dataset. (**B**) The response rate to immunotherapy in low and high groups stratified by disulfidptosis score in the melanoma dataset. (**C**) Kaplan-Meier curves for OS in late-stage non-squamous non-small cell lung cancer combined targeted therapy (bevacizumab + erlotinib) comparing low and high groups stratified by the disulfidptosis score. (**D**) The response rate to combined targeted therapy (bevacizumab + erlotinib) in late-stage non-squamous non-small cell lung cancer in low and high groups stratified by disulfidptosis score. (**E**, **F**) Bubble plot showing the relationship between mRNA expression of genes related to disulfidptosis in pan-cancer and GDSC or CTRP drug sensitivity (top 30). The color from blue to red represents the correlation between mRNA expression and IC_50_. Blue bubbles represent negative correlations; red bubbles represent positive correlations; the deeper the color, the higher the correlation. A positive correlation means that a gene with a high level of expression is resistant to the drug, and vice versa. The bubble size positively correlates with the FDR significance.

As the above findings indicate that the immune microenvironment is in an immunosuppressive condition when the disulfidptosis score is high, we hypothesized that patients with high disulfidptosis scores would have poorer immunotherapeutic efficacy. Therefore, we investigated the association between the disulfidptosis score and the sensitivity of patients to immunotherapy. We found that the immunotherapy response rate was lower in the high disulfidptosis group than that in the low disulfidptosis group in the melanoma dataset (Figure 9A, 9B). Subsequently, we also observed that late-stage non-squamous non-small cell lung cancer combined with targeted therapy (bevacizumab + erlotinib) in the high disulfidptosis score group had a lower response rate than that in the low disulfidptosis score group (Figure 9C, 9D).

We further analyzed the correlation between the mRNA expression of genes related to disulfidptosis in pan-cancer and drug sensitivity in the Genomics of Drug Sensitivity in Cancer (GDSC) and the Cancer Therapeutics Response Portal (CTRP) (top 30). We found that high expression of NCKAP1, RAC1, and SLC7A11 and low expression of WASF2 were associated with resistance to chemotherapy for several GDSC or CTRP small molecules (Figure 9E, 9F).

## DISCUSSION

The discovery of disulfidptosis and its related mechanisms presents interesting potential targets for the manipulation of this new form of cell death and highlights the possibility of finding new therapies and target identification for cancer. Given the appealing role of disulfidptosis in tumors, it may be highly important to explore disulidptosis in many types of cancer. However, no pan-cancer studies have been conducted to investigate the role of disulfidptosis in various malignancies. In our investigation, we thoroughly examined disulfidptosis genes in multiple tumors and datasets.

The current investigation showed the expression landscape of disulfidptosis-related genes in tumor and normal tissues from 33 malignancies. In 31 malignancies, we discovered that the expression of these genes differed considerably between tumor and normal samples. Compared to normal samples, certain tumors showed higher expression, whereas others showed lower expression. In our study, SLC7A11, RPN1, and RAC1 were highly expressed in most tumors. Higher expression levels of NCKAP1, SLC3A2, and WASF2 were observed in CHOL, GBM, LIHC, STAD, ESCA, LGG, PAAD, and THYM tissues. Furthermore, positive correlations between the expression levels of the disulfidptosis-related genes indicated that they may share some roles or functions. We also found that disulfidptosis-related genes predicted patient survival in certain cancer contexts. Subsequently, altered levels of the disulfidptosis score expression were found to be related to patient prognosis by Cox proportional hazard regression model analysis and Kaplan-Meier analysis.

Furthermore, the association between disulfidptosis and various pathway scores was identified via ssGSEA in 33 cancer types. We discovered that disulfidptosis is closely related to immune-related pathways, including TGF beta signaling, NOTCH signaling, angiogenesis, and WNT beta catenin signaling. We then studied the relationship between the disulfidptosis score and the tumor microenvironment in a variety of tumors. The results indicated an obvious negative relationship between the immune score and most cancers. The immunological score, as we know, measures the fraction of immune cells infiltrated in tumor tissues. In addition, the relationship between the disulidptosis score and immune cell infiltration in various cancers was investigated.

In the majority of tumors, we discovered a negative correlation between the disulfidptosis score and anti-tumor immune cells, such as CD8+ T cells, Tgd, and NK cells. CD8+ T lymphocytes are regarded as key participants in the fight against tumors [17]. The activation of T gamma delta (Tgd) cells is regulated by a number of cellular and molecular elements, including cytokine signals and stress ligands produced by tumors. They are used in cancer immunotherapies and play a crucial role in tumor immunosurveillance because they may react to oncogenic transformation quickly (within seconds to minutes, as opposed to days) [18] without the need for clonal expansion. They have the capacity to stimulate the production of inflammatory mediators, such as IFN- and TNF, destroy tumor cells, and link the innate and adaptive immune systems [18, 19]. By regulating the activity of other innate and adaptive immune populations in the TME, NK cells can indirectly control tumor growth [20] in addition to directly controlling tumor growth through direct contact between NK cells and tumor cells.

We also discovered a positive correlation between the disulfidptosis score and immune cells such as Treg cells, Th17 cells, and neutrophils, which are important in pro-tumor immunity. As they can inhibit anti-tumor immune effector responses in the TME by producing IL-10 and TGF-β, regulatory T cells (Tregs) are thought to support the survival of the TME. Tregs are characterized by immunosuppressive and tolerogenic functions in both homeostasis and inflammation [21]. At present, patients with giloblastoma (tumor recurrence and poor prognosis) [22] and CRC [23] have been described as having a role for Tregs in immunosuppressive processes. Th17 cells have been linked to a poor prognosis in a number of malignancies, including colorectal, breast, and prostate tumors [24–26]. Furthermore, it can trigger angiogenesis in human malignancies such as gastric [27], pancreatic [28], lung [29], and colorectal [30]. Moreover, it can prevent tumor cell death, encourage tumor spread, and impede effective antitumor therapy [31]. The pro-tumor activity of neutrophils has been linked to the development of tumors through the expression of prostaglandin E2 [32], stimulation of tumor growth by TNF-induced IL-17-producing CD4+T cells [33] and matrix metalloproteinase 8 or 9 [34]. All of these findings indicate that patients with a high disulfidptosis score are immunosuppressed. Consistent with this conclusion, we found that patients with higher disulfidptosis scores had poorer efficacy in the melanoma dataset. We also found that high expression of disulfidptosis-related genes is associated with resistance to most drugs or small molecules in the Genomics of Drug Sensitivity in Cancer (GDSC) and Cancer Therapeutics Response Portal (CTRP) projects.

In conclusion, we provided a thorough and in-depth assessment of the roles of disulfidptosis-related genes in expression, survival, drug resistance, and the tumor microenvironment, which is extremely valuable as a new target for clinical diagnosis and treatment. Our study has some limitations. First, other independent databases did not independently verify the findings of our study; hence, our future work will involve verifying them using our data and other public databases. A more significant point is that this study’s data were based on bioinformatics analysis and did not include *in vitro* or *in vivo* experimental findings. Therefore, we intend to use molecular and animal studies to provide further insight into the function of genes associated with disulfidptosis in cancer.

## MATERIALS AND METHODS

### Data collection

Genotype-Tissue Expression (GTEx) data was downloaded from Xena, and RNA expression data was downloaded from the Cancer Genome Atlas (TCGA).

### Disulfidptosis genes differential expression analysis

SangerBox software was used to compare mRNA expression levels of disulfidptosis genes between tumors and normal tissues for various cancer types. Cancer and paracancerous samples from 33 different TCGA cancer types were compared (Supplementary Table 1), and comparable normal samples from the GTEx database were added for secondary comparison because of the minimal number of paracancerous tissues in the TCGA database. The expression of the disulidptosis gene in cancer and non-cancer tissues was compared using a t-test.

### Evaluation of the disulfidptosis score

The disulfidptosis score was calculated based on single-sample gene-set enrichment analysis (ssGSEA) [35] using the disulfidptosis-related genes to quantify the expression levels of these genes in each cancer. We estimated the disulfidptosis score of 33 cancers from the TCGA database.

### Genetic alteration analysis

Using the “Quick Selection” and “TCGA Pan-Cancer Atlas Study” tools on the cBioPortal website (https://www.cbioportal.org/), genetic variant characterization of disulfidptosis genes was established. Additionally, the “Cancer Type Summary” module of the TCGA database was used to query the mutation frequency, copy number variation, and mutation type.

### Survival analysis

The link between the disulfidptosis score and overall survival (OS), progression-free survival interval (PFI), and disease-specific survival (DSS) was investigated using multivariable Cox regression analysis using the TCGA database. For each cancer type, p-values and hazard ratios (HR) with 95% confidence intervals (CI) were calculated.

### Gene set variation analysis

Gene set variant analysis (GSVA) was performed on all TCGA samples using the HALLMARK pathway database from the molecular signatures database (MSigDB).

### Immunohistochemistry (IHC) staining

IHC images of healthy liver tissues and LIHC tissues showing the protein expression of disulfidptosis-related genes were retrieved from the HPA (Human Protein Atlas) (http://www.proteinatlas.org/) and examined to assess variations in disulfidptosis-related gene expression at the protein level.

### Cell culture

BEAS-2B, H226, H1975, H1299, Huh7, Hep3B, SNU475 and THLE-3 were purchased from the Cell Bank of the Chinese Academy of Sciences and routinely maintained at 37° C in a 5% CO2 incubator with the recommended culture medium.

### RNA isolation and qRT-PCR

TRIzol Reagent (Invitrogen, China) was used to extract total RNA from tissues and cells, and a Reverse Transcription Kit (Applied Biosystems, USA) was used to reverse-transcribe cDNA.

The SYBR Green Kit (Vazyme, China) and Roche LightCyclerTM 480 were used in accordance with the manufacturer’s instructions to perform qRT-PCR. The 2^-ΔΔCT^ method was used to examine the data. The endogenous control was β-actin. The primers used in qRT-PCR have been listed in Supplementary Table 2.

### Western blot analysis

Total cell line protein was extracted using RIPA Lysis Buffer and PMSF (Thermo Fisher Scientific, USA) according to the manufacturers’ instructions. After centrifuging at 13,000 g for 15 min, the supernatant was extracted for further study. The membrane was incubated with primary antibodies, including anti-SLC3A2 (1:1000 dilution, PA596401, Thermo Fisher), anti-NCKAP1 (1:3000 dilution, PA5-88433, Thermo Fisher), anti-SLC7A11 (1:1000 dilution, ab175186, Abcam), anti-WASF2 (1:1000 dilution, PA5-60975, Thermo Fisher), anti-RAC1 (1:1000 dilution, PA1-091, Thermo Fisher), and anti-RPN1 (1:2,000 dilution, ab197888, Abcam) and anti-GAPDH (1:10000 dilution, ab181602). Secondary antibodies and CA were purchased from Cell Signaling Technology (Danvers, MA, USA). The images were captured using the Gel Dox XR system (Bio-Rad, Philadelphia, PA, USA). The experiment was repeated three times, independently.

### Tumor microenvironment analysis

Based on TCGA expression data, “estimate” R packages were used to explore the immune score, estimate score, and stromal scores of various tumor patients. The association between these scores and the disulfidptosis score was determined using the Spearman test.

### Immune infiltrate analysis

The TIMER2 and ImmuCellAI databases were used to collect data on the relationship between the disulfidptosis score and immune infiltration. The Pearson correlation coefficient was used to determine the relationship between the disulfidptosis score and the number of several cell types, including CD4+ T cells, CD8+ T cells, neutrophils, macrophages, eosinophils, and natural killer cells. ESTIMATE was used to determine the relationship between the disulfidptosis score and the immune score., stromal score, and ESTIMATE score.

### Statistical analyses

The Mann-Whitney U-test was used to analyze the statistically significant differences between non-normally distributed variables, whereas the unpaired Student’s t-test was used to determine the statistical significance of the differences between regularly distributed variables. To compare more than two groups, a one-way ANOVA was utilized as a parametric alternative to the non-parametric Kruskal-Wallis test. The relationship between the mRNA expression of the disulfidptosis genes was examined using Spearman’s correlation. The R programming language (version 4.2.2) was used.

## Supplementary Material

Supplementary Figures

Supplementary Tables
