# Supplementary material for: Pan-cancer analysis of disulfidptosis with potential implications in prognosis, immune microenvironment, and drug resistance in human cancer
Source: Aging (Albany NY). 2024 Jul 3;16(13):10997–1017. doi: 10.18632/aging.205993 (PMC11272104; doi:10.18632/aging.205993)
Supplement: Supplementary Figures [file aging-16-205993-s001.pdf]

SUPPLEMENTARY FIGURES

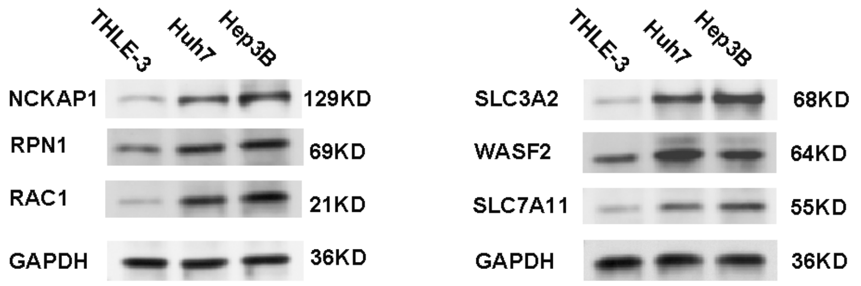

Supplementary Figure 1. Disulfidptosis genes' protein expression was detected in hepatocellular carcinoma cells and normal liver epithelial cells via Western-blotting analysis.

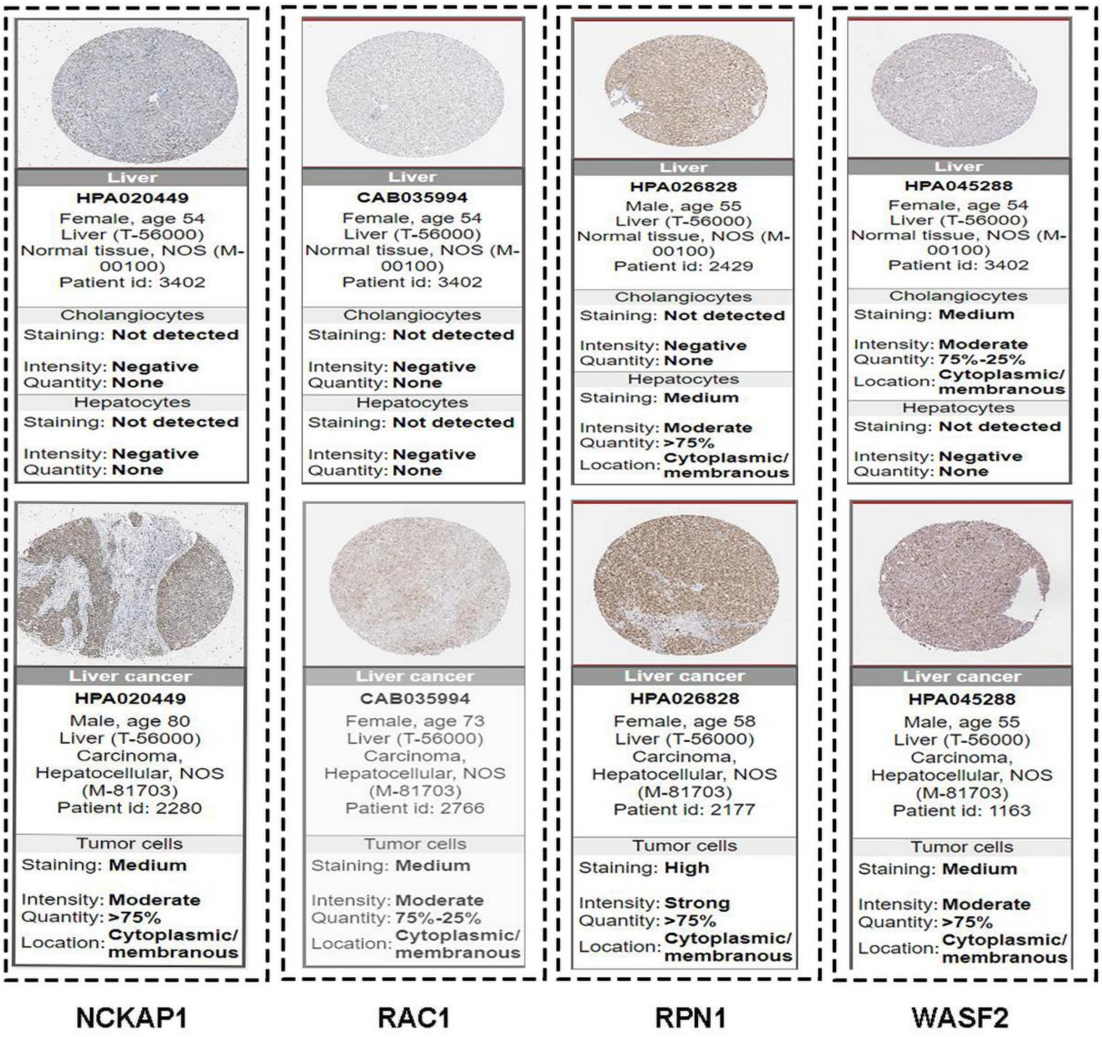

Supplementary Figure 2. Patient information and the detailed staining of immune-histochemistry pictures from the HPA database.

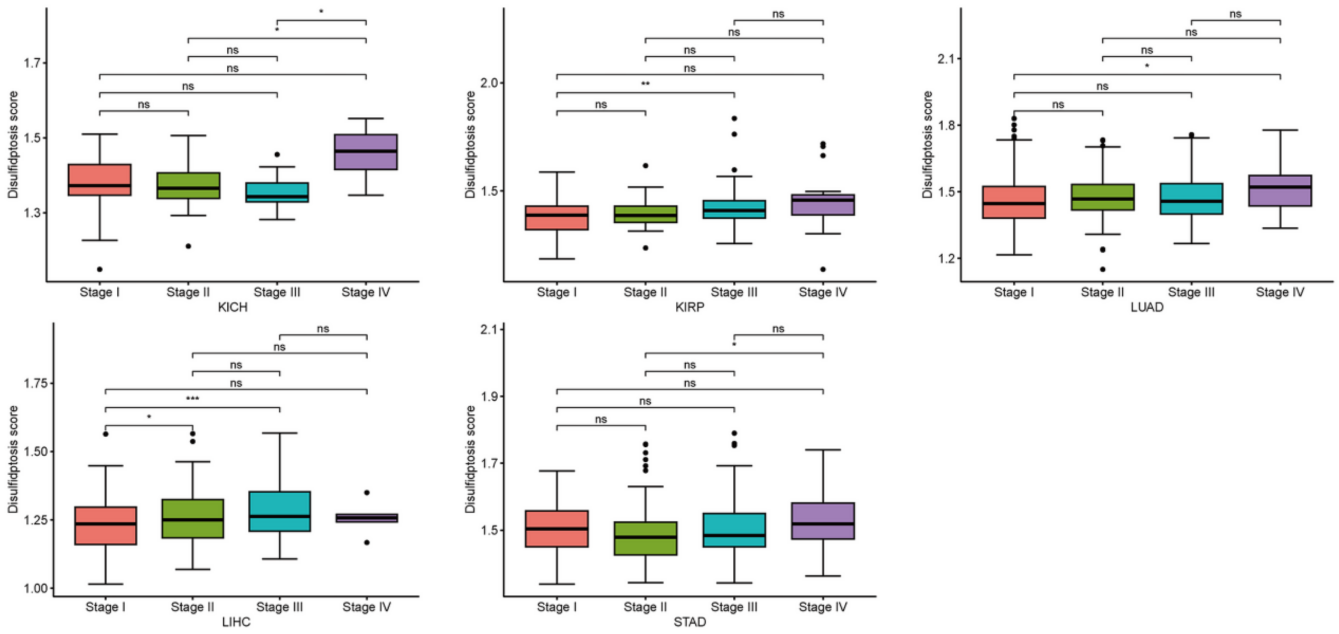

Supplementary Figure 3. Differences in the disulfidptosis score in different stages of different tumors. *p< 0.05, ** p< 0.01, and *** p< 0.001, ns, No statistical significant.
