# Supplementary material for: Pan-cancer analysis of disulfidptosis with potential implications in prognosis, immune microenvironment, and drug resistance in human cancer
Source: Aging (Albany NY). 2024 Jul 3;16(13):10997–1017. doi: 10.18632/aging.205993 (PMC11272104; doi:10.18632/aging.205993)
Supplement: Supplementary Tables [file aging-16-205993-s002.pdf]

SUPPLEMENTARY TABLES

Supplementary Table 1. List of cancer types analyzed in this study.

TCGA code	Cancer type	Histology
ACC	Adrenocortical carcinoma	Carcinoma
BLCA	Bladder urothelial carcinoma	Carcinoma
BRCA	Breast invasive carcinoma	Carcinoma
CESC	Cervical squamous cell carcinoma and endocervical adenocarcinoma	Carcinoma
CHOL	Cholangiocarcinoma (bile duct)	Carcinoma
COAD	Colon adenocarcinoma	Carcinoma
DLBC	Lymphoid neoplasm diffuse large B-cell lymphoma	Lymphoma
ESCA	Esophageal carcinoma	Carcinoma
GBM	Glioblastoma multiforme	Sarcoma
HNSC	Head and neck squamous cell carcinoma	Carcinoma
KICH	Kidney chromophobe	Carcinoma
KIRC	Kidney renal clear cell carcinoma	Carcinoma
KIRP	Kidney renal papillary cell carcinoma	Carcinoma
LAML	Acute myeloid leukemia	Leukemia
LGG	Brain lower grade glioma	Sarcoma
LIHC	Liver hepatocellular carcinoma	Carcinoma
LUAD	Lung adenocarcinoma	Carcinoma
LUSC	Lung squamous cell carcinoma	Carcinoma
OV	Ovarian serous cystadenocarcinoma	Carcinoma
PAAD	Pancreatic adenocarcinoma	Carcinoma
PCPG	Pheochromocytoma and paraganglioma (adrenal gland)	
PRAD	Prostate adenocarcinoma	Carcinoma
READ	Rectum adenocarcinoma	Carcinoma
SARC	Sarcoma	Sarcoma
SKCM	Skin cutaneous melanoma	
STAD	Stomach adenocarcinoma	Carcinoma
TGCT	Testicular germ cell tumors	Carcinoma
THCA	Thyroid carcinoma	Carcinoma
THYM	Thymoma	Lymphoma
UCEC	Uterine corpus endometrial carcinoma	Carcinoma
UCS	Uterine carcinosarcoma	Mixed type
UVM	Uveal melanoma	Carcinoma

Supplementary Table 2. The primer sequences used in this study.

Gene	Primer sequence (5' -> 3')	
NCKAP1	Forward Primer	TTGTACCCCATAGCAAGTCTCT
	Reverse Primer	GGGCATTTCTCCACTGGTCAG
RAC1	Forward Primer	ATGTCCGTGCAAAGTGGTATC
	Reverse Primer	CTCGGATCGCTTCGTCAAACA
RPN1	Forward Primer	GGCCAAGATTTCAGTCATTGTGG
	Reverse Primer	CTTCGTTGGATAGGGAGAGTAGA
SLC3A2	Forward Primer	TGAATGAGTTAGAGCCCGAGA
	Reverse Primer	GTCTTCCGCCACCTTGATCTT
SLC7A11	Forward Primer	TCTCCAAAGGAGGTTACCTGC
	Reverse Primer	AGACTCCCCTCAGTAAAGTGAC
WASF2	Forward Primer	TAGTAACGAGGAACATCGAGCC
	Reverse Primer	AAGGGAGCTTACCCGAGAGG
